# Impact of Wolfmet Tungsten Alloys as Parallel-Hole Collimator Material on Single-Photon Emission Computed Tomography Image Quality and Functional Parameters: A Simulating Medical Imaging Nuclear Detectors Monte Carlo Study

**DOI:** 10.1055/s-0043-1771287

**Published:** 2023-09-06

**Authors:** Maryam Darami, Babak Mahmoudian, Michael Ljungberg, Jalil Pirayesh Islamian

**Affiliations:** 1 Medical Radiation Sciences Research Team, Tabriz University of Medical Sciences, Tabriz, Iran; 2 Department of Nuclear Medicine, Faculty of Medicine, Tabriz University of Medical Sciences, Tabriz, Iran; 3 Medical Radiation Physics, Lund University, Lund, Sweden; 4 Department of Medical Physics, Faculty of Medicine, Tabriz University of Medical Sciences, Tabriz, Iran

**Keywords:** Wolfmet, collimator, image quality, SPECT, SIMIND, tungsten

## Abstract

**Objectives** Collimators have a significant role in image quality and detectability in single-photon emission computed tomography (SPECT) imaging. Using an appropriate alloy that effectively absorbs scattered photons, without induced secondary x-rays, and with proper rigidity and weight may provide an effective approach to the image improvement that conventionally collimators made of lead (Pb).

**Materials and Methods** A Siemens E.CAM SPECT imaging system equipped with low-energy high-resolution (LEHR) collimator was simulated by the Simulating Medical Imaging Nuclear Detectors Monte Carlo program. Experimental and simulated data were compared based on a 2-mm ^99m^ Tc point source in an acrylic cylindrical Deluxe phantom (Data Spectrum, Inc). Seven types of tungsten (W) alloys (Wolfmet), with W content from 90 to 97% by weight, were then used as collimator materials of the simulated system. Camera parameters, such as energy- and spatial resolution, image contrast, and collimator-related parameters, such as fraction of septal penetration, scatter-to-primary ratios, and percentage of induced secondary x-rays, due to interactions in the collimator, were evaluated.

**Results** Acceptable conformity was found for the simulated and experiment systems in terms of energy spectra, 10.113 and 10.140%, full width at half-maximum (FWHM) of the point spread function (PSF) curves, 8.78 and 9.06 mm, sensitivity, 78.46 and 78.34 cps/MBq, and contrast in images of 19.1 mm cold spheres in the Deluxe phantom, 79.17 and 78.97%, respectively. Results on the parameters of the simulated system with LEHR collimator made from the alloys showed that the alloy consisting of 90% W, 6% nickel, and 4% copper provided an FWHM of 8.76 mm, resulting in a 0.2% improvement in spatial resolution. Furthermore, all the Wolfmet collimators showed a 48% reduction in the amount of X-rays production compared to the Pb.

**Conclusion** A Wolfmet LEHR collimator, made by a combination of W (90%), Ni (6%), and Cu (6%) provides a better image quality and detectability compared to the Pb.

## Introduction

Single-photon emission computed tomography (SPECT) is a substantial tomographic functional imaging modality used to detect and characterize a variety of diseases, including cancers and infections. 1 2 SPECT is a cost-effective imaging modality 3 4 5 that improves diagnostic accuracy by facilitating three-dimensional information of the radiopharmaceutical distribution in in vivo and is also a preferable imaging approach due to radioisotope availability. However, SPECT images can be strongly influenced by penetration and scattering in the collimator walls and related X-ray emissions. Meanwhile, optimization of these effects may improve image quality and lesion detectability. 6 7 The purpose of a collimator is to let only those photons that propagate in an appropriate direction along a collimator hole to pass through and interact in the scintillation crystal but stop others. 8 9 However, not all photons will be stopped by the collimator walls. In order to improve collimator performance, the collimator absorption efficiency 10 11 can be increased by using materials of high atomic numbers and mass density. Conventional nuclear medicine collimators are fabricated from lead (Pb) with small amounts of antimony (Sb). Pb is considered as effective shielding material for impinging photons because of its high density (11.35 g/cm ^3^ ) and atomic number ( *Z* = 82). 12 13 In spite of other properties such as low cost and easy access, it is toxic, nonrigid, and produce secondary ionizing radiations of relatively high energies; 14 15 Pb may not be a proper collimator material of imaging systems, at least when it is used on its own.

In addition to Pb, other materials that have been used or proposed as collimator materials are tungsten (W), gold (Au), uranium (U), and platinum (Pt). However, because of substantial costs, Pb and W are by far the most commonly used materials. 16

In a study by Rajaee et al, on the effect of Au, Pb, U, and W as a high-energy-general-purpose parallel-hole collimator material on image quality, they demonstrated that the spatial resolution improved as the atomic number increased but with a cost of the system sensitivity. 10 Lee et al have shown from Monte Carlo simulations that the use of U provided about 3.19, 4.19, and 8.01% higher spatial resolution compared to Au, W, and Pb, although the system sensitivity of Pb was determined 4.25, 6.53, and 10.28% compared to the W, Au, and U, respectively. 17 In 2013, Weng et al, showed that imaging systems with a W parallel-hole collimator and cadmium zinc telluride (CZT) detectors compared to using Pb with a conventional NaI(Tl) detector have reduced the amount of septal penetration, image noise, and sharpness, and they concluded that the reduction may be attributed to W linear attenuation coefficient and also better energy resolution of CZT detector. 18 In another study by Lee et al, it was shown that the spatial resolution of U, with the same sensitivity, was better than Pb, W, and Au. 11 In this regard, it was suggested that the Pb–Sb alloy with a ratio of 98 and 2%, compared to the common Pb collimator, improves spatial resolution and energy resolution about 1.3 and 3%, respectively, with equal sensitivity. 11 They have also reported that the Pb–Sb alloy collimator improved the contrast and detection capability of the simulated cold and hot liver lesions of an NURBs based cardiac and torso (NCAT) Human Torso Phantom compared to the Pb alone.

In studies on metal alloys as pinhole collimator materials, Peterson et al used Rose's metal alloy, an alloy consisting of bismuth (Bi), Pb, and tin (Sn) with fractional weights of 50, 28, and 22%, respectively, in preclinical small‐animal imaging. Their studied parameters included penetration-to-scatter and penetration-to-total component ratios, system sensitivity, and the spatial resolution for two radionuclides, ^99m^ Tc and ^125^ I. 19 The authors concluded that Rose's metal is an alternative to standard materials not only for low-energy photon imaging but also for medium-energy applications that require low-cost, flexible pinhole configurations and designs, and that can tolerate a degraded spatial resolution compared to Au and W.

Due to its high density of 19.26 g/cm ^3^ and the atomic number of 74, W exhibits excellent absorption behavior against photon radiation such as X-rays and γ-radiation. 20 In an experimental study on shielding features of W–Cu, with weighting percentages of W85 wt%–Cu15 wt % and W75 wt%–Cu25 wt% composites, at low energy 0.266 MeV, Dong et al have found highest values of linear attenuation coefficient, 2.11 and 4.33 cm ^−1^ for W75 wt%–Cu25 wt% and W85 wt%–Cu15 wt%, while they found the lowest values with high-energy 1.25 MeV x-rays, 0.44 and 0.48 cm ^−1^ for W75 wt%–Cu25 wt% and W85 wt%–Cu15 wt%, respectively, so it is concluded that W–Cu composites are suitable to be used in several applications of radiation protection. 21

Kaur et al presented in a review article the applicability of metallic alloys including W, Cu, Fe, Ni, and Pb-based alloys in γ-rays shielding and expressed this in terms of mass-attenuation coefficient (μ _m_ ), effective atomic number (Z _eff_ ), electron density (Ne), mean-free path (mfp), half-value layer (HVL), tenth-value layer (TVL), exposure buildup factor, and energy exposure buildup factor, and they concluded that mass-attenuation coefficient values of Pb-based alloys and W-based alloys with lowest values of mfp, HVL, and TVL are comparable because they present high atomic numbers in a large weight percentage ratio than the other constituent elements. However, both the melting point and cost factor are high for W-based alloys. 22 Murty et al evaluated Z _eff_ of different W–Cu alloys by exposure to photons with an energy range of 60–400 keV. They extended the study to also check the validation of the mixture rule and any energy dependency of effective atomic number in the energy range of 100 to 1,400 keV. Both studies showed no energy dependence of Z _eff_ . 23 24

W alloy performs close to Au in terms of stopping power, with a better trade-off between performance and cost with ^131^ I imaging. 25 In 2019, Nguyen et al compared the performance of Pb, W, Au, and depleted U pinhole collimators in terms of spatial resolution on EXIRAD-3D, a newly autoradiography technique based on a highly focusing multipinhole collimator that achieves micronresolution SPECT for cryo-cooled tissue samples, for different radioisotopes including ^111^ In (171 keV and 245 keV), ^99m^ Tc (140 keV), ^201^ Tl (71 keV), and ^125^ I (27 keV). 26 They found that the image contrast-to-noise ratio (CNR) was improved with a higher atomic number in a range from 1.9 to 36.6%. They suggested W collimator to be a good choice to use for a wide ranges of SPECT radioisotopes. 26

**Fig. 1:**
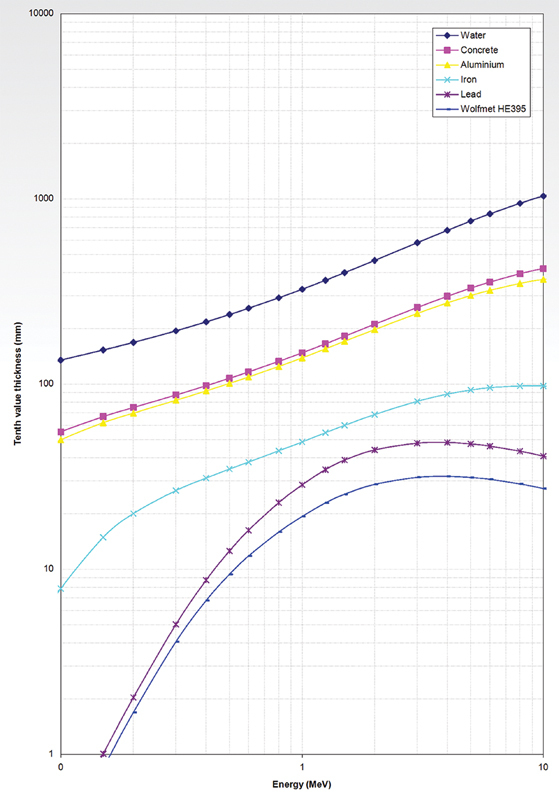
Comparative absorption data (10th value layer) of water, concrete, aluminum, iron, lead, Wolfmet ^®^ HE 395, as a function of the energy of incident narrow-beam photons. Data supplied by the National Physical Laboratory. 27

W alloys, including Wolfmet, do not only have a very high mass density, but are very good in attenuating ionizing radiation. 27 This makes the alloys ideal for shielding proposes in nuclear medicine or nuclear industry. Fig. 1 demonstrates the photon absorption characteristics of Wolfmet HE 395, a composite of W (95%)–Ni (3.5%)–Fe (1.5%), and other shielding materials. The TVL is plotted as a function of photon energy assuming narrow beam exposure condition. The data were supplied by the National Physical Laboratory. 27 HE 395 and Pb have shown TVLs of 24.3 and 38.3 mm, against _60_ Co gamma rays (1,173.2 and 1,332.5 keV), with HVL thickness of 7.0 and 11.7 mm, respectively.

Here, we evaluated the effects of Wolfmet tungsten alloys, as the LEHR collimator material, on the functional parameters and the image quality of an SPECT system.

## Material and Methods

We used the Simulating Medical Imaging Nuclear Detectors (SIMIND) Monte Carlo program (developed by Prof. Michael Ljungberg, Lund University, Lund, Sweden) to simulate a Siemens E.CAM SPECT camera (Siemens Medical Solutions, Erlangen, Germany). 28 29 In brief, each of the two camera heads consisted of a removable low-energy high-resolution (LEHR) collimator, a thin NaI(Tl) scintillation crystal, a light-guide, and an array of photomultiplier tubes (PMTs). The geometry of LEHR collimator was included parallel hexagonal holes defined as 1.11-mm flat-to-flat hole diameter, 24.05 mm hole length, and 0.16-mm septal thickness. The NaI (Tl) crystal was rectangular in shape with the dimension of 59.1 × 44.5 × 0.95 cm. The light yield was 40k photons/MeV with a peak emission spectrum at 415 nm. 7 29 The emitted light-photons from the various interactions in the NaI (Tl) crystal were collected by 59 PMTs where 53 of them had a diameter of 7.6 cm and the other six had a 5.1 cm diameter. The photocathode was of the bialkali type with a quantum efficiency of approximately 30%. The light guide with coupling grease ensured an effective light collecting and a good optical coupling between the scintillating crystal and PMTs to reduce nonlinearity and so contribute to spatial resolution. To consider the contribution of various structures attached to back of the crystal on backscattering of the emitted photons, mainly with high-energy gamma rays, a single slab of Pyrex was also substituted and included in the simulation. 30

Experimental data were obtained to validate the SIMIND simulations and these were based on scanning (1) a 2 mm ^99m^ Tc point source with 3.7 MBq activity located at a distance of 10 cm from the detector, and (2) a clear Acrylic Plexiglass Deluxe ECT (model ECT/DLX/P) water-filled cylindrical phantom with cold spheres and rods ( Table 1 ) in a hot-background using 370 MBq ^99m^ Tc. 31 The energy window was defined as 126 to 154 keV.

**Table 1:** Specification of the Deluxe phantom

Interior cylinder dimensions	216 mm ø x 186 mm
Cylinder wall thickness	3.2 mm
Cylinder volume	6.75 L
Cylinder volume with inserts	6.1 L
Cold rods insert height	88 mm
Sphere center from base plate	127 mm

Based on NEMA specifications on quality control procedures, the system spatial and energy resolution were determined from planar images of ^99m^ Tc point source acquiring at a distance of 10 cm from the collimator surface and center of the field of view. Matrix size was 128 × 128 matrix size with 0.27 mm pixel size, and 10 million counts per projection were collected. 32 33 Additionally, the system sensitivity (cps/MBq) specified for a point source at 25 cm from the surface of the detector.

Image contrast and spatial resolution were evaluated by simulating the SPECT imaging of a digitized cylindrical Deluxe phantom. 31 In brief, SPECT projections along the axis of rotation were calculated for a situation with the phantom centrally positioned at 15 cm distance from the collimator surface using a matrix size of 128 × 128 with 0.39 cm pixel size, 128 projection views in a 360 degrees clockwise rotation with one million counts per projection. An OS-EM reconstruction algorithm was used to reconstruct the SPECT projections with eight iterations and four subsets. Compensation for photon attenuation was included. The spatial resolution and image contrast were determined visually from the smallest recognizable rods and 19.1 mm spheres in the reconstructed images, respectively. 7

Simulations were conducted using the conventional LEHR collimator design with two Pb materials and multiple Wolfmet tungsten alloys ( Table 2 ).

**Table 2:** Details on the Wolfmet alloys components as a LEHR collimator 35

Collimator material	Composition (weight %)
Lead	Pb (100%)
Lead antimony	Pb (98%), Sb (2%)
Tungsten	W (100%)
W_ha190	W (90%), Ni (6%), Cu (4%)
W_ha193	W (93%), Ni (3.5%), Cu (3.5%)
W_ha195	W (95%), Ni (2.5%), Cu (2.5%)
W_he390	W (90%), Ni (5%), Fe (5%)
W_he395	W (95%), Ni (3.5%), Fe (1.5%)
W_he397	W (97%), Ni (1.5%), Fe (1.5%)
W_he3925	W (92.5%), Ni (3.8%), Fe (3.8%)

Abbreviation: LEHR, low-energy high-resolution.

## Results

**Fig. 2:**
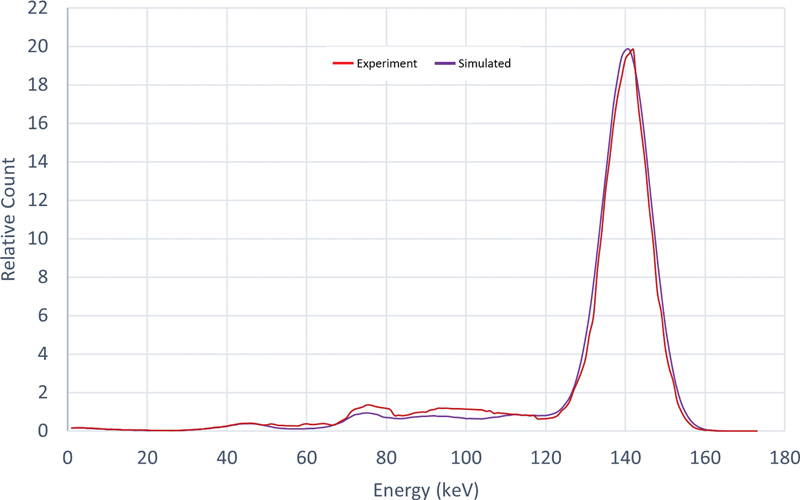
Energy pulse-height distribution obtained from the ^99m^ Tc point source (140 keV) simulated by SIMIND ( *blue* ) and from measurement ( *red* ).

A comparison of measured and simulated energy pulse-height distributions yields an acceptable conformity in terms of the functional and image parameters ( Fig. 2 ).

The calculated energy resolution (FWHM) from the ^99m^ Tc point source from simulation and measurement were calculated to 10.11 and 10.14%, respectively. The spatial resolution (FWHM) at 10 cm source to collimator distance was calculated to 8.78 and 9.06 mm for simulation and measurement and the system sensitivity was determined to be 78.46 and 78.34 cps/MBq, respectively.

Contrast measures from the cold sphere images of cylindrical Deluxe phantom were calculated according to the following equation:



where M _sp_ and M _cy_ correspond to the minimum pixel value of the cold spheres and the maximum pixel value in the Deluxe phantom cylinder, respectively. The results on contrast values of cold bodies in background of the Deluxe phantom cylinder are presented in Table 3 .

**Table 3:** Results on the calculated image contrast of Deluxe phantom cold spheres

System	Diameter of the Deluxe phantom cold spheres (mm)					
	9.5	12.7	15.9	19.1	25.4	31.8
Measurement	53.24	59.79	69.44	78.97	87.39	92.47
Simulation	51.92	56.48	68.89	79.17	83.03	89.21

**Fig. 3:**
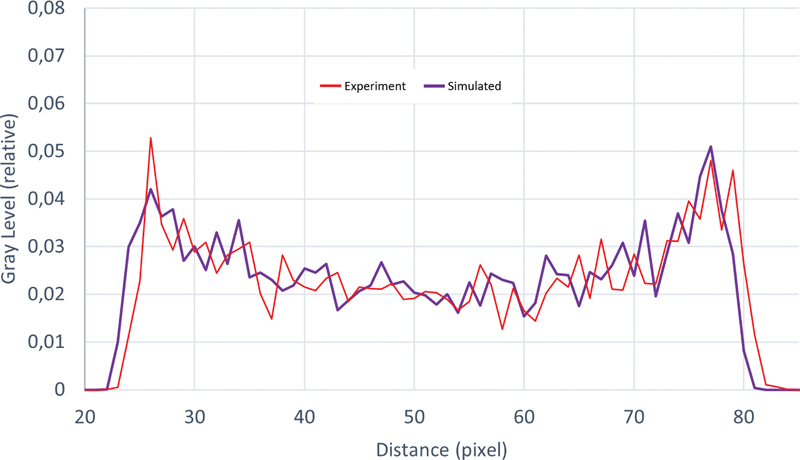
Profiles of image uniformity obtained from mages of Deluxe phantom SPECT from measurement ( *blue* ) and simulation ( *red* ).

Profiles on image uniformity, without attenuation correction as with the experimental, obtained from images of the Deluxe phantom are presented in Fig. 3 for the experiment and simulated SPECT imaging systems.

Results on the functional parameters of the simulated SPECT system equipped with 10 collimator materials from the ^99m^ Tc point source simulations are presented in Table 4 .

**Table 4:** Functional parameters of the simulated SPECT system from the ^99m^ Tc point source scanning with 10 different collimator materials

Material a	Sensitivity (cps/MBq)	Efficiency		Spatial resolution (mm)	
		Window	Spectrum	FWHM	FWTM
Pb	78.459	0.7388	0.9336	8.780	15.52
Pb_Sb	78.635	0.7390	0.9334	8.807	15.58
W	77.233	0.7534	0.9323	8.751	15.42
W_ha190	78.266	0.7531	0.9321	8.762	15.50
W_ha193	77.927	0.7536	0.9324	8.771	15.51
W_ha195	77.824	0.7531	0.9325	8.781	15.48
W_he390	78.388	0.7535	0.9322	8.800	15.52
W_he395	77.629	0.7537	0.9322	8.766	15.48
W_he397	77.533	0.7535	0.9324	8.772	15.48
W_he3925	78.041	0.7531	0.9322	8.791	15.50

Abbreviation: FWHM, full width at half-maximum.

a Pb (lead), Sb (antimony), W (tungsten).

Results on the collimator-related parameters, including septal penetration, geometric photons, Compton scatter in collimator, and the ratio of the scattered-to-primary photons in the photopeak energy windows, besides, the percentage of the collimator-induced x-rays are shown in Table 5 . The parameters were calculated by SIMIND during the simulation according to the related system, isotope, and phantom settings and, hence, were presented as data in the result files.

**Table 5:** Radiation interaction events of a 2 mm ^99m^ Tc point source with 3.7 MBq activity with the simulated SPECT system depend on collimator materials

Collimator material	Penetration (%)		Primary (%)		Scatter (%)		c X-Ray (%)	Scatter/Total ×10 ^−5^
	AC a	WEW b	AC	WEW	AC	WEW	AC	Total
Pb	6.21	4.62	87.62	93.95	2.20	1.43	3.96	535
Pb_Sb	6.29	4.73	87.57	93.84	2.21	1.44	3.93	536
W	3.88	3.42	91.15	94.16	1.81	1.44	2.05	538
W_ha190	4.79	4.27	92.93	95.60	1.97	1.57	2.09	536
W_ha193	4.54	4.02	91.44	94.45	1.94	1.53	2.09	538
W_ha195	4.38	3.87	91.60	94.62	1.91	1.51	2.11	536
W_he390	4.80	4.27	91.12	94.17	1.98	1.56	2.10	535
W_he395	4.29	3.83	91.76	94.67	1.87	1.88	2.07	536
W_he397	4.15	3.69	91.90	94.82	1.86	1.48	2.09	536
W_he3925	4.57	4.07	91.37	94.40	1.95	1.54	2.10	535

Abbreviation: SPECT, single-photon emission computed tomography.

a Photons after collimator.

b Photons within energy windows.

c X-ray in collimator was equal zero for all collimators.

**Fig. 4:**
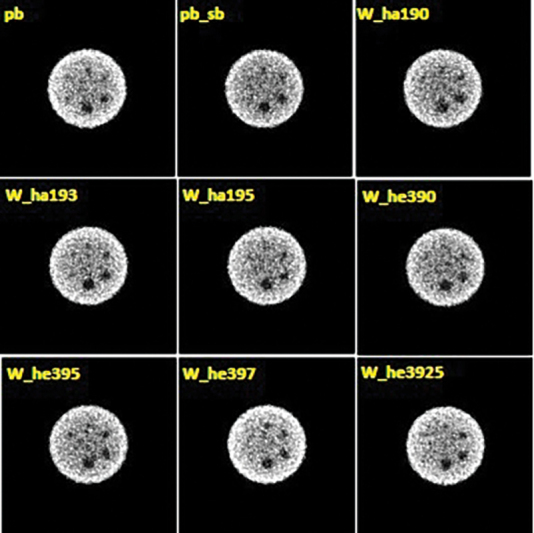
Reconstructed images of the SIMIND simulated SPECT of cold spheres (slice 56) of Deluxe phantom by with the LEHR collimator made from lead (Pb), lead–antimony (Pb–sb), tungsten (w), and Wolfmet alloys. SPECT projections along the axis of rotation were calculated for a situation with the phantom centrally positioned at 15 cm distance from the collimator surface using a matrix size of 128 × 128 with 0.39 cm pixel size. 128 projection views in a FO clockwise rotation with 1M counts per projection. A OSEM reconstruction algorithm was used to reconstruct the SPECT projections with eight iterations and four subsets. Compensation for photon attenuation was included.

Table 6 provides the measures of image contrast of the Deluxe phantom produced from the simulated SPECT system with different collimator materials. Reconstructed images of the cold spheres using the collimators are presented in Fig. 4 .

**Table 6:** The results on the SPECT image contrast of 19.1 mm cold sphere of Deluxe phantom with different collimator materials

Collimator material	Pb	Pb_Sb	W	W_ha190	W_ha193	W_ha195	W_he390	W_he395	W_he397	W_he3925
contrast (%)	79.17	79.25	77.97	79.00	80.49	79.15	78.76	78.76	80.33	80.18

Abbreviation: SPECT, single-photon emission computed tomography.

## Discussion

The purpose of the present study was to investigate the effects of W alloys as collimator material of SPECT imaging system on functional parameters of the system including spatial resolution, sensitivity, and so image quality by SIMIND Monte Carlo simulation.

In this regard, seven alloys of Wolfmet tungsten were used in the simulation of the potential benefits of LEHR W collimators for the current SPECT imaging system ( Table 2 ). According to the results, W alloys collimators demonstrated less septal penetration, scatter radiation, and also a better primary ratio compared to the Pb which produces secondary ionizing radiations with relatively high energies especially at whole the energy spectra ( Table 5 ). Furthermore, tungsten with a high density and atomic number of 19.26 g/cm ^3^ and 74, respectively, may exhibit excellent absorption behavior against the photon radiations and hence on image contrast ( Table 6 ).

Verification of the simulation with the conventional LEHR collimator confirmed the results of Azarm et al 29 34 and Toossi et al. 29 Meanwhile, according to the obtained results, there were 1.40 and 1.18% reduction in system sensitivity with W_he397 alloy, compared to Pb–Sb and Pb alone ( Table 4 ), respectively. Azarm et al, in a study on the effects of collimator material on spatial and energy resolution, have shown that the common collimator made of 98% Pb and 2% Sb could provide a better spatial and energy resolution, 7.68 mm and 9.87%, compared to 7.74 mm and 10.14% of Pb alone, respectively. 34 Our study also showed that Pb–Sb collimator improved the spatial resolution by 0.31% compared to Pb, and W_ha190 alloy further improved to about 0.2% compared to Pb and Sb. It must be mentioned that all the studied alloys showed a reduction in the penetration fraction, collimator-induced X-rays, and increased geometrical fraction compared to Pb. However, Collimator with W alone provides better results for the parameters compared to the studied materials ( Table 5 ). In this regard, results on the evaluation of pinhole collimator materials for micronresolution ex vivo SPECT by Nguyen et al 2019 indicate that using materials with higher photon attenuation including U, Au, W, and Pb yields images with better CNR for the ^125^ I, ^201^ Tl, ^99m^ Tc, and ^111^ In isotopes with improvements ranging from 1.9 to 36.6%. 26 They have suggested W collimator as a good choice for a wide range of SPECT isotopes, so our results also confirmed their findings. In a similar manner with photon-stopping power in terms of photon attenuation, increasing the linear attenuation coefficient was demonstrated to have potential effects on the improvement of the spatial resolution and, hence, on isotopic image quality, for example, image contrast, resolution, and detectability. 11 17 It should be noted that linear attenuation coefficient is also influenced by the beam energy. 21 22 Regarding image contrast, the value obtained for the 19.1 mm cold sphere inside the Deluxe phantom increased by 1.32, 1.16, and 1.01 with W_ha193, W_he397, and W_he3925 compared to the Pb, respectively ( Table 6 and Fig. 4 ). One explanation could be the penetration rate reduction.

Finally, the emergence of new production techniques, such as direct 3-D printing of metals or “cold casting” of W-composite materials, will open new possibilities for the fabrication of more tailored collimator designs that would be impossible or very expensive to construct by conventional methods of fabrication including casting hot Pb (microcast) or folding Pb foil (microlinear).

## Conclusion

The present study showed that the use of a potential LEHR collimator made from Wolfmet tungsten alloys could a better spatial resolution, improved contrast, and image quality in general when comparing conventional Pb and Pb–Sb collimators.
